# Comprehensive Diagnosis, Treatment, and Outcome of *Taenia crassiceps* Cysticercosis in a Ring-Tailed Lemur (*Lemur catta*) from a Croatian Zoo: No Longer Unusual?

**DOI:** 10.3390/pathogens13040283

**Published:** 2024-03-27

**Authors:** Lea Grbavac, Ana Šikić, Petar Kostešić, Ivan-Conrado Šoštarić-Zuckermann, Vesna Mojčec Perko, Jadranko Boras, Ingeborg Bata, Andrija Musulin, Tara Kostanjšak, Tatjana Živičnjak

**Affiliations:** 1 Parasitology and Invasive Diseases Unit, Faculty of Veterinary Medicine, Heinzelova 55, 10 000 Zagreb, Croatia; akajmic@vef.unizg.hr (A.Š.); tatjanaz@vef.unizg.hr (T.Ž.); 2 Clinic for Surgery, Orthopaedics and Ophthalmology, Faculty of Veterinary Medicine, Heinzelova 55, 10 000 Zagreb, Croatia; pkostesi@vef.unizg.hr (P.K.); amusulin@vef.unizg.hr (A.M.); 3 Veterinary Pathology Unit, Faculty of Veterinary Medicine, Heinzelova 55, 10 000 Zagreb, Croatia; isostaric@vef.unizg.hr; 4 Microbiology and Infectious Diseases Unit, Faculty of Veterinary Medicine, Heinzelova 55, 10 000 Zagreb, Croatia; vmojcec@vef.unizg.hr; 5 Zagreb Zoo, Fakultetsko dobro Street 1, 10 000 Zagreb, Croatia; jadranko.boras@zoo.hr (J.B.); inga.bata@zoo.hr (I.B.); 6 Fitzpatrick Referrals, Halfway Lane, Eashing, Godalming GU7 2QQ, UK; tara.kostanjsak@gmail.com

**Keywords:** *Cysticercus longicollis*, *Taenia crassiceps*, metacestode, subcutaneous, ring-tailed lemur, formalin-fixed and paraffin-embedded tissue, PCR, Croatia

## Abstract

*Taenia crassiceps* is a zoonotic tapeworm of the genus *Taenia* that is distributed throughout the Northern Hemisphere. Wild and domestic carnivores are final hosts, while rodents and rabbits are primarily intermediate hosts, although many other mammals may harbour the larval stage, *Cysticercus longicollis*. This case report aims to describe *C. longicollis* infection in a lemur and molecularly characterise the isolated parasite. The excised lesion was subjected to morphological and histopathological examination, which revealed cysticerci of the tapeworm. Formalin-fixed and paraffin-embedded block (FFPEB), as well as the cysticerci fixed with formalin stored for one year, were subjected to molecular analysis, which aimed at detecting the partial mitochondrial cytochrome c oxidase subunit 1 (cox1) gene of *Taenia* sp. Based on the morphological characteristics, the parasite was identified as a metacestode of *T. crassiceps*. The presence of the cox1 gene was detected using polymerase chain reaction (PCR) in all samples. A randomly selected PCR product was sequenced and compared with other sequences from the GenBank database, confirming that the detected parasite was *T. crassiceps.* This article reports the first case of *T. crassiceps* cysticercosis in a lemur (*Lemur catta*) in Croatia and emphasises the potential risk of transmission from wild carnivores.

## 1. Introduction

Tapeworms are flat, segmented endoparasites that belong to the class Cestoda, within the phylum Platyhelminthes. Within this class, there are 18 orders, with the Cyclophyllidea and Diphyllobothriidea orders being particularly significant in human and veterinary medicine [1,2]. Cyclophyllidea encompasses a diverse group of tapeworms that have an indirect life cycle and primarily infect terrestrial vertebrates. This order includes the Taeniidae family, whose adults live in the small intestine of many carnivorous mammals and humans. Although adult tapeworms usually do not cause noticeable clinical symptoms, serious complications such as ileus or intestinal perforation may arise in cases of high parasite burden [3,4]. Similarly, the clinical manifestations of infection with larval stages of tapeworms depend on factors such as the number, size, intermediate host, and location of the parasite and can be potentially life threatening [2].

Tapeworms of the Taeniidae family are unique in that they require mammals as their final and intermediate hosts [5]. Larval stages or metacestodes develop in various tissues and organs of intermediate hosts, causing metacestodoses such as hydatidosis caused by the larval stage of the genus *Echinococcus*, coenurosis, and cysticercosis caused by certain species of the genus *Taenia*, which have major medical and veterinary impacts [5,6,7,8]. The sources of infection for intermediate hosts are embryonated eggs in the faeces of the final host, as well as contaminated food or water. On the other hand, the final hosts become infected by ingestion of organs and tissues that contain larval stages of tapeworms [1].

The determination of tapeworm species based on morphological criteria is a complex task that requires extensive expertise [9,10,11]. However, the use of molecular methods provides a more reliable approach to determining specific species of tapeworm and discovering previously unknown species [5,12,13]. For that purpose, various genetic markers are analysed, with a particular emphasis on sequencing different genes found in mitochondrial DNA (mtDNA) [14,15].

*Taenia crassiceps* (Zeder, 1880) is a zoonotic tapeworm of the genus *Taenia* that is distributed throughout the Northern Hemisphere. The adult tapeworm lives in the small intestine of wild and domestic carnivores, with the red fox (*Vulpes vulpes*) being the most common final host in Europe [16]. Natural infections with *T. crassiceps* have also been described in arctic foxes (*Vulpes lagopus*), sand foxes (*Vulpes ferrilata*), wolves (*Canis lupus*), golden jackals (*Canis aureus*), raccoons (*Procyon lotor*), raccoon dogs (*Nyctereutes procyonoides*), wild cats (*Felis silvestris*), stone martens (*Martes foina*), domestic dogs (*Canis lupus familiaris*), and cats (*Felis catus*) (reviewed in [17]). Although rodents and rabbits are primarily intermediate hosts, numerous other mammals, including humans, can also harbour the larval stage [6,17,18,19]. In intermediate hosts, upon ingestion of a taeniid egg, an oncosphere, also called a hexacanth embryo, hatches from the egg, penetrates the intestinal wall, and migrates through the bloodstream or lymph flow to the tissues or organs of predilection, such as the subcutis, muscles, and body cavities. Here, the oncosphere grows, differentiates, and forms a cyst-like larval stage, *Cysticercus longicollis.* The cysticercus is transparent, varies considerably in size (1–11 mm in diameter), and consists of a fluid-filled bladder with a single scolex invaginated or evaginated at one pole. The scolex is equipped with four suckers and two rows of large and small rostellar hooks [16,20]. *C. longicollis* exhibits a unique ability to reproduce asexually and form many cysticerci by exogenous and endogenous budding, resulting in continuous and uncontrolled proliferation [16]. As a consequence, severe cysticercosis can develop in intermediate hosts, leading to a fatal outcome. Several studies reported that carnivores such as dogs, cats, and foxes can also develop cysticercosis caused by *C. longicollis*, presuming that immunocompromised status was the main cause of disease occurrence [19,20,21,22,23,24].

Humans and non-human primates may serve as dead-end hosts, obtaining *T. crassiceps* cysticercosis by consuming food and water contaminated with taeniid eggs excreted in the faeces of the final host [17]. Several cases of *T. crassiceps* cysticercosis in humans have been reported in Canada, the USA, Germany, France, and Switzerland, as reviewed in [17]. The larval stages of the parasite have been found in subcutaneous tissue, muscles, eyes, and the brain, with molecular confirmation provided in some cases [17,25,26,27]. While most infections have been observed in immunocompromised individuals [17,28,29,30,31], there have been some rare cases of infection in immunocompetent individuals [25,26,27]. Cysticercosis caused by *T. crassiceps* has also been reported in captive non-human primates in zoos [17]. Ring-tailed lemurs (*Lemur catta*) have been found to be infected with other metacestodes in addition to *C. longicollis* [32,33,34,35,36,37,38,39], indicating a high susceptibility to infection with larval stages of tapeworms in lemurs in general [17].

In Croatia, cysticercosis caused by *T. crassiceps* in a red fox has recently been recorded [21]. However, there is a lack of available data on the prevalence of this tapeworm in final or intermediate hosts. In this study, the first case of subcutaneous cysticercosis caused by *T. crassiceps* in a ring-tailed lemur (*Lemur catta*) from Zagreb Zoo in Croatia is presented, and the article includes a molecular characterisation of the isolated parasite.

## 2. Materials and Methods

### 2.1. Case Description

This study focused on an adult female ring-tailed lemur (*Lemur catta*) born on 2 March 2009 at Zagreb Zoo in Croatia. The animal had been living in a group consisting of its father (born in 1998), a twin brother (born in 2009), and a younger sister (born in 2013). They had been living in a den whose indoor enclosure covered a total of 70 m^2^, while the outdoor enclosure extended over 500 m^2^. The outdoor enclosure was delineated by eight rows of electric wires and lacked a roof structure. In a certain area of indoor enclosure, visitors could interact directly with the resident animals. Zagreb Zoo is located in Maksimir Park (3.2 square kilometres), which, despite being located in the urban area, serves as an important habitat for numerous free-living wild animals, including red foxes (*Vulpes vulpes*), martens (*Martens martens*), and red squirrels (*Sciurus vulgaris*).

In March 2021, a fluctuating 3 × 3 cm subcutaneous mass was observed on the chest of the aforementioned lemur. To further investigate this abnormality, the animal was captured within its enclosure and sedated on site using a combination of the following anaesthetics: 0.04 mg/kg of medetomidine (Domitor 1 mg/mL solution for injection, Orion Corporation, Espoo, Finland) and 0.1 mg/kg of midazolam (Dormicum 15 mg/3 mL solution for injection, Cheplapharm Arzneimittel GmBH, Greifswald, Germany) administered intramuscularly (i.m.). A fine needle aspiration (FNA) was performed to obtain a sample, which was then sent to a board-certified veterinary pathologist for cytological examination. The findings indicated a cystic structure filled with proteinaceous fluid and a purulent necrotic content. In the absence of other cellular elements, it was not possible to determine the origin of the structure.

Examination of the sedated animal revealed swelling of the vulva, whereupon a smear sample was taken and subjected to bacteriological analysis. Results showed dense growth of *Corynebacterium* sp. The animal was treated with oral suspension of amoxicillin/clavulanic acid (Augmentin^®^ powder for oral suspension 400 mg/57 mg/5 mL, GlaxoSmithKline, Dublin, Ireland) at a dose of 15 mg/kg bid for 7 days. No further clinical signs were observed. In addition, a stool sample was collected for coprological parasitological examination, which was negative. As the cyst on the chest was not large and cytology was undetermined, a benign mass was suspected, and it was decided not to operate.

Since the cyst on the chest had increased in size over time, it was decided to surgically remove it one year after its initial description. In February 2022, the animal was captured in its enclosure, sedated again with the same anaesthesia protocol, and transferred to the Surgery Clinic of the Faculty of Veterinary Medicine for the procedure.

### 2.2. Surgical Procedure and Sampling

Upon arrival at the Surgery Clinic on the 10th of February 2022, the vein catheter was inserted and the animal was induced in anaesthesia using propofol 10 mg/kg (Propofol 1% (10 mg/1 mL), Fresenius Kabi Austria GmbH, Graz, Austria). Analgesia was provided with fentanyl at a dose of 0.1 μg/kg/min CRI (Fentanyl 50 μg/mL solution for injection, Piramal Critical Care B.V., Voorschoten, The Netherlands) and 0.2 mg/kg of meloxicam i.m. (Meloxidolor 5mg/mL, Dechra, Bladel, The Netherlands).

**Figure 1:**
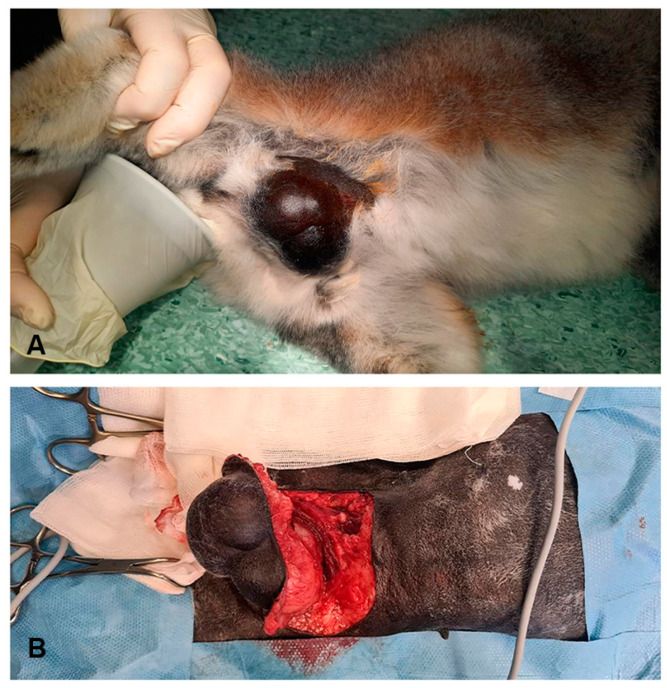
Subcutaneous cyst on a ring-tailed lemur’s chest. A cyst before surgery (**A**). Excision of the lesion compressing the subcutaneous tissue and *m. pectoralis* (**B**).

The excision of the cyst on the animal’s chest was performed under general inhalation anaesthesia with sevofluarane (Sevorane, AbbVie S.r.l., Campoverde di Aprilia, Italy) in the supine position. The cyst was 5 × 4 cm in size and exhibited soft elastic consistency. It compressed the subcutaneous tissue and fibres of the pectoralis muscle (*m. pectoralis*) extending into the deeper tissue layers (Figure 1). Manipulation of the cyst led to its rupture, releasing numerous small cysts. Following the cyst wall rupture, a saline lavage was performed and the remaining cyst wall and numerous cysts were subsequently removed. The tissue around the cyst was surgically debrided. The affected area was reconstructed with a monofilament resorbable suture in three layers: the fascia was reconstructed with simple interrupted sutures, following the simple continuous suture of the subcutis and dermis (Biosyn 3-0 and Biosyn 4-0, Covidien, Ireland).

Two hours after the surgical procedure, the animal recovered consciousness and was returned to its group. Over the next 7 days, the animal received meloxicam (Meloxoral 1.5 mg/mL oral suspension for dogs, Dechra, Bladel, The Netherlands) at a dose of 0.1 mg/kg and pradofloxacin (Veraflox 5 mg/mL oral suspension for cats, Bayer, Leverkusen, Germany) at a dose of 5 mg/kg perorally. All lemurs received albendazole (Alphaben 100 mg/mL, AlphaVet^®^, Budapest, Hungary) at a dose of 25 mg/kg twice daily perorally for 14 days in two rounds. Two years later, at the time of writing this article, none of the lemurs showed any clinical signs of primary disease.

The excised lesion was fixed in 4% formaldehyde and sent to a board-certified veterinary pathologist for histological examination. Additionally, the material released when the cyst ruptured was collected, fixed in 4% formaldehyde, and sent for morphological examination to the Parasitology Unit of the Faculty of Veterinary Medicine in Zagreb, after which the sample was stored in an archive.

### 2.3. Sample Preparation and DNA Extraction

DNA extraction was carried out on a one-year-old archived sample of cysticerci fixed in 4% formaldehyde and formalin-fixed paraffin-embedded blocks (FFPEB) of the subcutaneous cyst. Samples were washed and deparaffinised prior to DNA extraction.

#### 2.3.1. Pretreatment of Formalin-Fixed Sample with Ethanol

Cysticerci stored for one year in 4% formaldehyde in a 50 mL conical tube at 4 °C were rinsed twice for 30 s with phosphate-buffered saline (PBS), transferred to another 50 mL tube with 0.9% NaCl solution, and rehydrated at 4 °C for 4 days. Cysticerci were then separated from the 0.9% NaCl solution with filtration through a gauze and transferred to two 1.5 mL microcentrifuge tubes, one containing 25 mg (F1) and the other 35 mg (F2) samples. Cysticerci were then washed three times in descending ethanol concentrations (100%, 75%, and 50%). For each wash, 500 μL of ethanol was added to the samples, briefly vortexed, and centrifuged at 700 rcf for 2 min, and excess ethanol was removed by pipetting. After the final wash and removal of excess ethanol, the cysticerci were air-dried overnight in a horizontal position. The next day, samples were stored at +4 °C until DNA extraction.

#### 2.3.2. Pretreatment Dewaxing of Formalin-Fixed and Paraffin-Embedded Tissue Block (FFPEB)

A year-old FFPEB of the subcutaneous cyst was cut into eight slices with a thickness of 8 μm using a rotary microtome (Microm HM 355 S, Thermo Fisher Scientific, Walldorf, Germany) and placed in two sterile 1.5 mL microcentrifuge tubes, each containing four slices. Two different dewaxing methods were used for each sample.

Deparaffinization of the first sample (P1) was performed with xylene according to the protocol described by Shi et al., 2002 [40]. After dewaxing with xylene and washing in 100% ethanol, the sample was air-dried overnight in a 1.5 mL microcentrifuge tube in a horizontal position and stored the next day at +4 °C until DNA extraction.

The second sample (P2) was deparaffinised using a microwave according to a published method [41]. To a 1.5 mL microcentrifuge tube containing the sample, 400 µL of 1× PBS was added, briefly vortexed, and centrifuged at 10,000 rcf for 3 min. Four holes were drilled in the lid of the microcentrifuge tube using a sterile needle. The tube was placed in a microwave on a foam tube rack and heated three times at 550 W for 15 s, then at 400 W for 10 s, and a second time for 15 s. After gentle vortexing, assuring the sample did not spill through the lid holes, and centrifugation at 10,000 rcf for 1 min, the supernatant was pipetted and discarded. The sample was then subjected to the DNA extraction procedure.

#### 2.3.3. DNA Extraction and Assessment of DNA Concentration and Purity

DNA was extracted from all four samples using a NucleoSpin Tissue Kit (Macherey-Nagel, Düren, Germany), according to the manufacturer’s instructions. Positive and negative controls were included in the extraction procedure. The concentration and purity of obtained DNA samples were analysed using the NanoDrop One spectrophotometer (Thermo Scientific, Waltham, MA, USA). In addition, all DNA samples were electrophoresed on a 1% agarose gel.

### 2.4. Polymerase Chain Reaction (PCR)

To detect the partial sequence of the mitochondrial cytochrome c oxidase subunit 1 gene (cox1) of the genus *Taenia*, a conventional PCR was performed on a T100 Thermal Cycler (Bio-Rad, USA) using primers (JB3 5-TTTTTTGGGCATCCTGAGGTTTAT-3 and JB4.5 5-TAAAGAAAGAACATAATGAAAATG-3′) and the protocol previously described by Bowles et al., 1992 [42]. In addition, negative (nuclease-free water) and positive control (*Echinococcus granulosus* template DNA) were included in the PCR. The PCR was performed as follows: 95 °C for 2 min; 40 cycles at 94 °C for 30 s, 53 °C for 30 s, 72 °C for 45 s, followed by 72 °C for 7 min. Amplification of PCR products was confirmed by electrophoresis (Consort EV232 Electrophoresis Power Supply, Consort, Turnhout, Belgium) in 1% agarose gel. Electrophoresis results were visualised using a UV transilluminator (Molecular Imager Gel Doc XR+, Bio-Rad, Hercules, CA, USA). An amplified fragment of 446 base pairs in each sample was considered a positive result.

### 2.5. Sequencing and Phylogenetic Analysis

A randomly selected PCR product from a formalin-fixed sample was sent to a commercial company (Macrogen, Amsterdam, The Netherlands) for sequencing to determine the species of *Taenia* tapeworm. Received sequences were edited in software programmes: BioEdit (version 7.7.1), Chromas (version 2.6.6), ClustalX2 (version 2.1), and Mega 11. The GenBank database was used for comparison of the sequencing results with the BLAST search engine. Evolutionary analyses were conducted in Mega 11.

## 3. Results

### 3.1. Parasitological Findings

**Figure 2:**
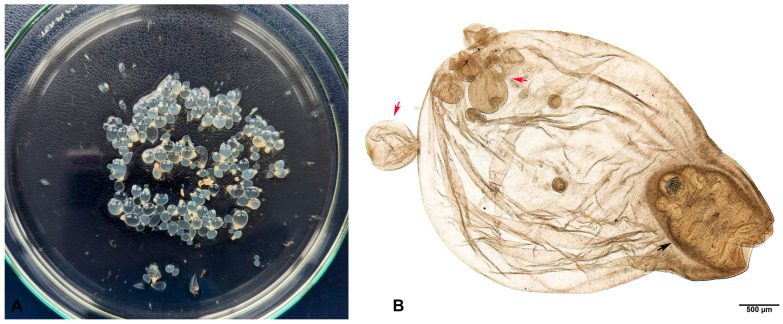
Metacestodes of *T. crassiceps* collected during rupture of the subcutaneous cyst from ring-tailed lemur’s chest. Cysticerci with unipolar white dots (scolices) (**A**). *C. longicollis* with invaginated scolex (black arrow) and exogenous budding (red arrows) (**B**).

The material collected from the ruptured cyst revealed transparent tapeworm cysticerci of oval-to-round shape and variable size (1–7 mm in diameter) with visible white dots representing invaginated scolices (Figure 2A). Each cysticercus had an invaginated scolex with four suckers and two rows of rostellar hooks. Single or multiple exogenous daughter buds were also observed in some cysticerci (Figure 2B). The parasite was identified as a metacestode of *T. crassiceps*, *C. longicollis*, based on morphological characteristics consistent with the description provided by Freeman, 1962 [16].

### 3.2. Histological Findings

**Figure 3:**
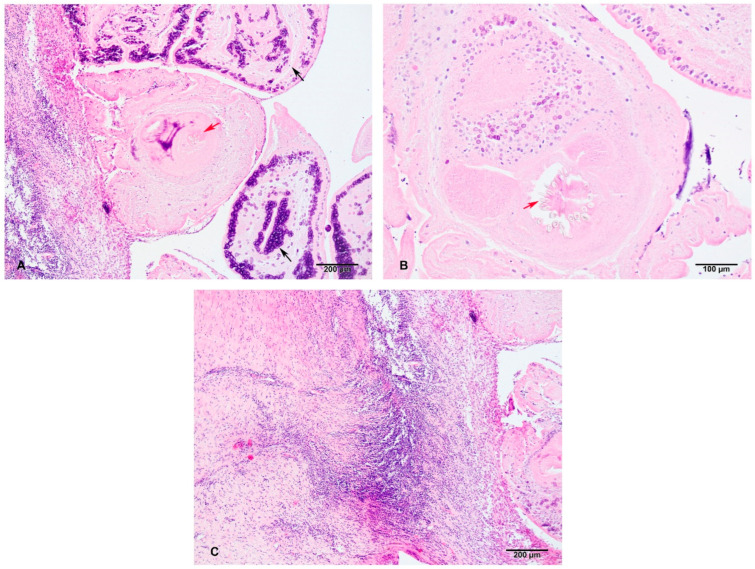
Microscopic findings: cysticerci and accompanying inflammation. Within the subcutis, there are several encapsulated cysticerci containing cross-sections of larval cestodes. These are characterised by a ridged tegument and fibrillar, eosinophilic, spongy parenchyma with numerous subtegumental oval basophilic calcareous corpuscles about 5 µm in diameter (black arrows). The central cross-cut of cysticercus reveals scolex with rostellum and birefringent hooklets (red arrow). The capsule is composed of still maturing fibrous tissue and some lymhoplasmacytic inflammatory infiltrate. H&E, 100× total magnification (**A**). Another scolex with better visualization of the rostellum with prominent birefringent hooklets (red arrow). H&E, 200× total magnification (**B**). Fibrous capsule surrounding the cysticerci (at right). The layers of the fibrous capsule at left contain more collagenous tissue and are mature, while the ones closer to the cysticerci are still immature (with activated fibroblasts) and contain focally extensive dense infiltrate of lymphocytes and fewer macrophages and plasma cells. Just next to the degenerating cysticercus, there is a narrow layer of epithelioid macrophages and one multinucleated giant cell (granulomatous inflammation). H&E, 100× total magnification (**C**).

In the examined skin sample, several dozen encapsulated cysticerci that extended from the superficial dermis deep into the subcutis with a total diameter of about 2–3 cm were observed. Cysticerci were surrounded by a fibrovascular capsule of varying thickness and maturity (Figure 3A,C). In several places, this capsule has ruptured, causing pronounced lymphoplasmacytic and histiocytic (including visible multinucleated giant cells) and, less frequently, eosinophilic inflammation. This inflammation penetrated the interior of the cyst at the rupture sites. The cysticerci consisted of a serrated tegument with fibrillar eosinophilic and spongy parenchyma, containing numerous basophilic calcareous bodies measuring 5–20 micrometres in diameter. In some cysticerci, scolices with a rostellum and hooks with double refraction of light were observed (Figure 3A,B). Most cysticerci were degenerated and infiltrated with marked histiocytic and lymphoplasmacytic inflammation as aforementioned. Furthermore, there was extensive focal dermal and subcutaneous granulomatous (histiocytic), fibrotic and lymphoplasmacytic inflammation, accompanied by numerous cysticerci.

### 3.3. Molecular Findings

**Figure 4:**
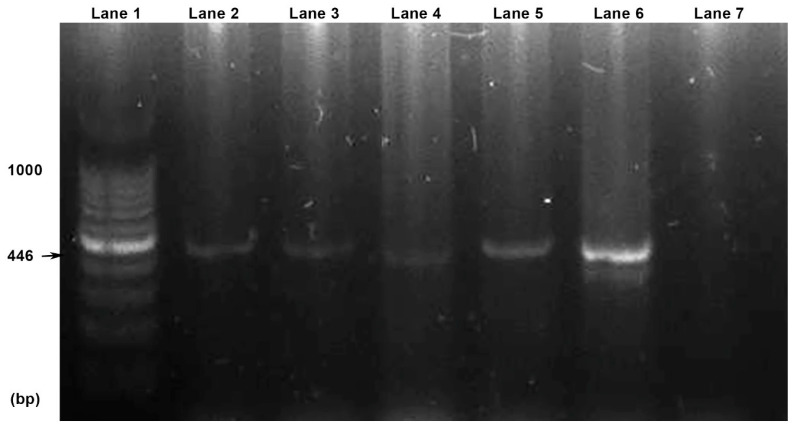
PCR reactions visualised by electrophoresis. Lanes: 1, 100 bp DNA ladder; 2, F1 sample; 3, F2 sample; 4, P1 sample; 5, P2 sample; 6, positive control; 7, negative control.

The concentrations and purity of all DNA samples were favourable for PCR (Table 1). All DNA samples were visible in electrophoresed 1% agarose gel. The presence of the cox1 gene with 446 bp was proven in all four samples using PCR (Figure 4). Comparison of the edited sequence with other sequences from the GenBank database identified the tapeworm as *T. crassiceps*. Our sequence was added to the GenBank database under accession number OR578398 (417 bp) and was compared to sequences available in the GenBank database with a length greater than 400 bp and with determined nucleotides (KY321321.1; KY321319.1; OR350516.1; OR350515.1; OM996999.1; KY321320.1; OK284537.1; OK284538; AF216699; MN514031; KY321318; MT806358; KY883633; AB033411; OP831183). The sequence showed 100% identity with the sequences with accession numbers KY321321 (*T. crassiceps*, Czech Republic, *Galago senegalensis*), KY321319 (*T. crassiceps*, Czech Republic, *Canis lupus familiaris*), OR350516 (*T. crassiceps,* Italy, *Lemur catta*), OR350515 (*T. crassiceps,* Italy, *Lemur catta*) and OM996999 (*T. crassiceps,* Poland, *Lemur catta*). The average base composition of the partial cox1 gene was A (22.3%), T (47%), G (22.5%), and C (8.2%). The average frequency number of codons was 136.

**Figure 5:**
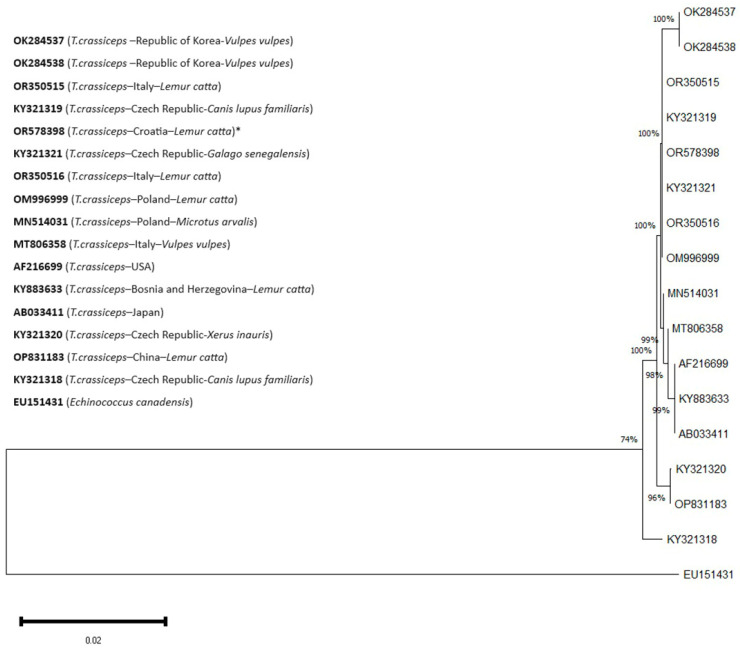
A phylogenetic tree of sequences with partial cox1 gene with their accession numbers. The percentage of replicate trees in which the associated taxa clustered together in the bootstrap test (1000 replicates) is shown next to the branches. *—sequence from this study. *E. canadensis*—outgroup.

Phylogenetic analysis revealed minimal variation compared to the sequences mentioned above, showing an identity range between 99.52 and 100%. As can be seen in the phenogram (Figure 5), our sequence of *T. crassiceps* genotype belongs to the same group as the sequences of *T. crassiceps* isolated from the following animals: *Galago senegalensis* (Czech Republic, KY321321.1), *Canis lupus familiaris* (Czech Republic, KY321319.1), *Lemur catta* (Italy, OR350516.1), *Lemur catta* (Italy, OR350515.1), and *Lemur catta* (Poland, OM996999.1). *Echinococcus canadensis* (accession number EU151431.1) was used as an outgroup [34,43]. The tree was drawn to scale, with branch lengths in the same units as those of the evolutionary distances used to infer the phylogenetic tree.

Codon positions included were 1st+2nd+3rd+noncoding. The analysis involved 17 nucleotide sequences (one ours, one outgroup, and fifteen of the most similar nucleotide sequences found in GenBank), comprising a total of 417 positions in the final dataset.

## 4. Discussion

Recently, cysticercosis of *T. crassiceps* has been described more frequently in ring-tailed lemurs, an atypical intermediate host, in European zoos [34,35,36,37,38,39], many of which were fatal [36,37,38,39]. This article describes the first documented report of subcutaneous cysticercosis in a ring-tailed lemur in Croatia. In Bosnia and Herzegovina [37], Italy [38] and Austria [39], no subcutaneous lesions were observed, unlike the cases reported in Poland [34], Serbia [35] and Spain [36]. In this article, a case similar to those found in the latter countries is described. However, the lesions in our case manifested as exclusive subcutaneous infection, which was successfully treated surgically, resulting in complete recovery of the animal, which is consistent with the case in Poland [34].

In Croatia, a severe case of cysticercosis was recently reported in a red fox near Zagreb, indicating the presence of the parasite in the fox population [21]. Final hosts, such as red foxes, have been sporadically sighted in Maksimir Park and in the zoo itself.

Considering that the lemur enclosure has an open area fenced with electric wire, foxes could have access to this part of the enclosure and contaminate the soil with taeniid eggs. Lemurs often engage in soil exploration within the open area, presumably in search of worms and other edibles, suggesting that the animal most likely acquired the parasite through contaminated soil. Despite the fact that lemurs receive their food in the enclosed part of the habitat, on a raised platform, it cannot be excluded that the potential source of infection was contaminated food (vegetables and fruit), as shown in Basel Zoo and Sarajevo Zoo [37,44]. It is worth mentioning that the case of fatal *T. crassiceps* cysticercosis described at the Sarajevo Zoo involved a lemur that originated from Zagreb and belonged to this captive-bred family. However, before the animal’s transfer to Sarajevo, the family lived in an older enclosure, fenced with bars that prevented foxes from entering. Therefore, the possibility of infection in Zagreb was ruled out in this case, as also stated by the authors [37].

In this study, parasite DNA was successfully extracted from formalin-fixed and paraffin-embedded (FFPE) samples and formalin-fixed samples that were stored for one year. Deparaffinization of FFPE using organic solvent xylene, as well as the microwave method, resulted in good yield and quality of the extracted DNA. The microwave method yielded less DNA than xylene, but the quality of the DNA remained similar. In contrast, some studies showed that the microwave method gave a higher yield of DNA compared to common organic chemical-based extraction methods [41,45]. Numerous protocols and commercial kits have been developed to facilitate molecular analysis of FFPE samples, as they represent a valuable resource not only for retrospective molecular research but also for routine clinical diagnostics, particularly in human medicine [41,46,47,48,49,50]. The microwave method is one of the simplest, most affordable, and time-saving techniques compared to other techniques, making it applicable in laboratories that are less equipped. Most parasite specimens in archives throughout the world are fixed in formalin for morphological analysis and permanent preservation. Although formalin fixation usually results in DNA fragmentation, modification, and nucleic acid cross-linking, which compromises the quality of DNA for molecular analysis, some studies provide efficient methods for the extraction of DNA from cestodes and other parasites preserved in formalin for a prolonged period of time [46,51,52,53,54,55]. Therefore, archived specimens preserved in formalin represent a promising resource for molecular analysis of older samples to accurately determine the species. The sequence obtained in this study showed 100% identity with sequences of *T. crassiceps* isolated from primates: Senegal bushbaby (*Galago senegalensis*) from the Czech Republic (KY321321), ring-tailed lemurs (*Lemur catta*) from Italy (OR350515, OR350516) and Poland (OM996999), and a dog (*Canis lupus familiaris*) from the Czech Republic (KY321319).

## 5. Conclusions

In this paper, the first appearance of *C. longicollis* infection in a non-human primate in Croatia has been described. The exact source of the infection was not proven in the current study, but given the presence of *T. crassiceps* in the fox population near Zagreb, this infection may be related. This work emphasised the need to test domestic and wild carnivores for this zoonotic tapeworm, as well as the risk of infection transmission between the zoo and wild animals where contact is possible. In future research, the prevalence of *T. crassiceps* in the red fox population in Croatia and in zoo carnivores should be investigated. In addition, this work highlights the possibility of molecular analysis of one-year-old formalin-fixed parasite samples, which should be considered for other similarly kept archival samples.

## Figures and Tables

**Table 1:** Concentration of DNA samples and purity from contaminating proteins (A260/A280), salts, and other contaminants (A260/A230).

Sample	Concentration (ng/µL)	A260/A280	A260/A230
F1	31.1	1.81	1.16
F2	48.5	1.88	1.57
P1	133.9	1.94	2.00
P2	71.9	1.90	1.81

## Data Availability

The partial mitochondrial cytochrome oxidase subunit 1 (cox1) gene sequence of *Taenia crassiceps* has been submitted to the NCBI database and is publicly available under the accession number OR578398.
